# Establishing functional lentiviral vector production in a stirred bioreactor for CAR-T cell therapy

**DOI:** 10.1080/21655979.2021.1931644

**Published:** 2021-05-28

**Authors:** Qu-Lai Tang, Li-Xing Gu, Yao Xu, Xing-Hua Liao, Yong Zhou, Tong-Cun Zhang

**Affiliations:** a Key Laboratory of Industrial Fermentation Microbiology of the Ministry of Education & Tianjin Key Laboratory of Industrial Microbiology, College of Biotechnology, Tianjin University of Science and Technology, Tianjin, China; b College of Life Science and Health, Wuhan University of Science and Technology, Wuhan, Hubei, China

**Keywords:** Functional, lentiviral vectors, HEK293T cells, stirred bioreactors, CAR-T cell therapy

## Abstract

As gene delivery tools, lentiviral vectors (LV) have broad applications in chimeric antigen receptor therapy (CAR-T). Large-scale production of functional LV is limited by the adherent, serum-dependent nature of HEK293T cells used in the manufacturing. HEK293T adherent cells were adapted to suspension cells in a serum-free medium to establish large-scale processes for functional LV production in a stirred bioreactor without micro-carriers. The results showed that 293 T suspension was successfully cultivated in F media (293 CD05 medium and SMM293-TII with 1:1 volume ratio), and the cells retained the capacity for LV production. After cultivation in a 5.5 L bioreactor for 4 days, the cells produced 1.5 ± 0.3 × 10^7^ TU/mL raw LV, and the lentiviral transduction efficiency was 48.6 ± 2.8% in T Cells. The yield of LV equaled to the previous shake flask. The critical process steps were completed to enable a large-scale LV production process. Besides, a cryopreservation solution was developed to reduce protein involvement, avoid cell grafting and reduce process cost. The process is cost-effective and easy to scale up production, which is expected to be highly competitive.

## Graphical abstract

**Figure:**
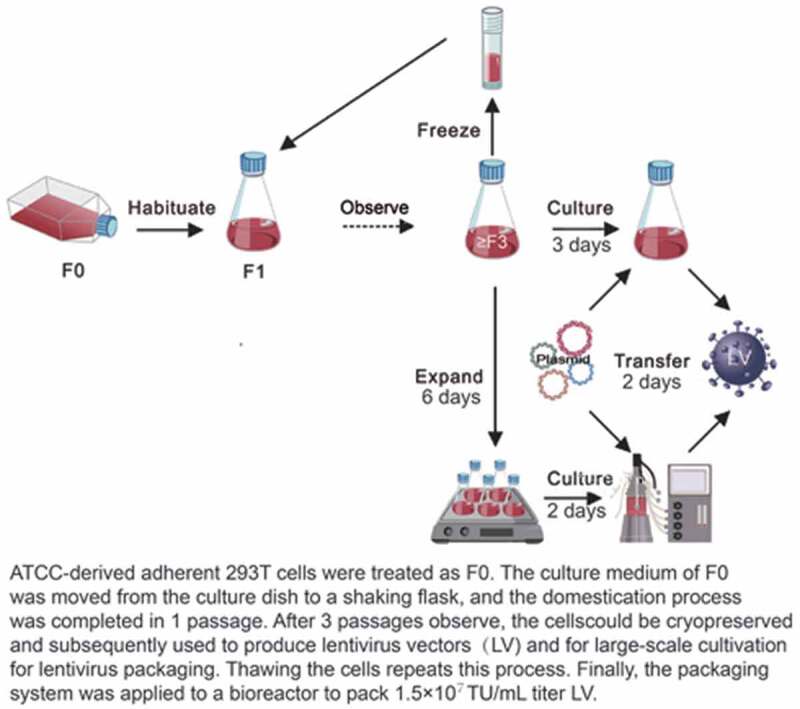

## Introduction

Chimeric antigen receptor therapy (CAR-T) is promising immunotherapy that can targeted destroy cancer cells. Presently, CAR-T is being used in clinical practice for blood cancers [1]. The core of CAR-T cells production is the production of LV [2,3]. Therefore, large-scale production and purification of high-titer LV become the key step for mainstream CAR-T cells manufacturers.

The traditional LV production process used adherent 293 T cells cultivated in the medium with serum [4]. However, the serum composition is complex and the batch difference of serum is unpredictable. Also, trypsin, used to process adherent culture, causes cell fragility, leading to partial membrane rupture and reducing the virus titer [5]; Most of the vaccines or LV in stationary culture systems are scaled up via cell factories and flasks, which are labor-intensive and susceptible to contamination [6].

Compared with adherent cells, suspension cells can be cultivated in a serum-free medium, clear and stable, and can thus avoid adverse cell interactions, simplify the upstream process, and achieve large-scale and high-density growth [7]. Ongoing development of serum-free medium cultivation is the current trend of mammalian cell culture technology [8]. The trend significantly improves the transition from laboratory to clinical-grade production. A previous study reported that the 293 T cell had been adapted in serum-free media as suspension cells using sequential adaptation approach [9]. However, using this approach with a long adaptation cycle [10], the proliferation of 293 T suspension cells was slower than original adherent cells, also LV yield. Considering the lentivirus packaging plasmids that are entirely dependent on the host proliferate for replication [11], therefore, it is very important to maintain cell proliferation, enhance its adapted efficiency and improve LV yield.

Stirred bioreactors have been widely used in the biopharmaceutical industry recently [12]. It is noteworthy that most of the 293 T cells are dependent on micro-carriers in the bioreactors [13] or efficient gene editing for virus production [14]. However, the cost of micro-carriers is high, and the process of cell sampling observation, culture, and large-scale production is complicated [15]. Gene editing technology allows gene inactivation [16] and maybe reduced titers [17]. A really suspension cells can grow freely and directly in the bioreactor and the culture environment is uniform, the operation is simple, the automation is feasible, and the pollution rate and costs are both low. At the same time, the cell digestion step is no longer needed, and the cell volume/density ratio is reduced. Suspension cells are easy to scale up to different containers, making automatic culture convenient while markedly reducing human intervention. Therefore, large-scale LV production is a significantly meaningful effort in suspension with stirred bioreactor without micro-carriers or gene editing.

Thus, the high-density culture of 239 T cell suspension in a stirred bioreactor without micro-carriers is a major challenge. Meanwhile, there is no previous research till date in the literature on the use of fast adaptation for really 293 suspension cells. The present study has been designed to adapt 293 T adherent cells during a short duration into suspension cells and maximize the production of functional LV in a 15-L 3-impeller agitating bioreactor. Our work provided a procedural reference for maximizing the large-scale clinical production of functional LV.

## Materials and methods

### Materials

Human renal epithelial cell line (293 T) was purchased from American Type Tissue Collection (CRL-3216). DMEM, fetal bovine serum (FBS), 0.25% trypsin-EDTA, and glutamic acid came from Gibco, (ThermoFisher Scientific, USA). 293 CD05 medium and OPM-CHO PFF06 medium (supplemented medium) were purchased from Shanghai OPM Biotechnology Co. Ltd. (Shanghai, China), which are serum-free, inorganic salts, amino acids, and vitamins; SMM293-TII was purchased from Sino Biological Inc. (Beijing, China) and is a serum-free medium designed for the cultivation of a mammalian cell type. Polybrene and polyethylenimine (PEI) were purchased from Sigma-Aldrich (St. Louis, MO, USA), and *Escherichia coli* DH5α was purchased from TIANGEN Biotech Co., Ltd. (Beijing, China). The expression plasmid (PTK-CAR-CD30-GFP), and packaged plasmid (pMDLg-pRRE, pRSV-REV, and pMD2.G) were provided by Wuhan Bio-raid Biotechnology Co., Ltd. (Wuhan, China). Except FBS, all materials were of non-animal origin in this study.

### Cell adaption

Our serum-free medium without antibiotics, comprised of 293 CD05 medium and SMM293-TII with 1:1 volume ratio, was named F medium (Supplemental material Table S1). Adherent 293 T cells were passaged after 3 days, using 0.25% trypsin-EDTA to detach cells from dishes, and counted by Trypan blue (Gibco) staining [18]. The cell survival rate was more than 90%. Those cells were centrifuged, resuspended with F medium, and inoculated at a density of 4.0 × 10^5^ cells/mL into a 20 mL/125 mL triangulation flask for cell adaption (direct method). The cells were cultivated at 37°C and 130 rpm on a shaking incubator (sk-r1807-e, SCTLOGEX, America) with 5% CO_2_. Cell morphology without shaking was observed using a bright field microscope and images were captured using a cell phone camera. Afterward, cells with shaking were sampled and counted every generation. The adaption criteria for 293 T suspension cells were the cell density from 4.0 × 10^5^ cells/mL to 1.6 × 10^6^ cells/mL after 3 days in culture, while cell viability of more than 90% was maintained [9,19].

### Preparation of suspension cell passages and growth curve determination

Suspension cells meeting indicators were grown for three generations and then cultured for 8 days with cell densities of 1.0 × 10^5^ cells/mL (group A), 2.0 × 10^5^ cells/mL (group B), and 4.0 × 10^5^ cells/mL (group C), respectively. Samples were taken every 12 hours to determine cell density and cell viability. We used a hemocytometer (717,805, Blaubrand, Germany) to record cell density. Cell viability was determined using Trypan blue staining.

### LV packaging verification

Four *Escherichia coli* DH5α cultures transfected with expressing plasmids and packaged plasmids were incubated overnight while oscillating. Then, the plasmids were extracted with a large plasmid extraction kit (QIAGEN, Germany). The four plasmid packaging systems were used to transfect suspension cells meeting indicators, and transfection was done with a final concentration of 6 µg/mL PEI and 2 µg/mL plasmid DNA (pMD2. G/pRSV-Rev/pRRE/PTK-CAR-CD30-GFP, 3:5:5:10 ratio). On transfection, the cell density was adjusted to 1 × 10^6^ cells/mL [20]. The 293 T suspension cells were cultured in shaking flasks and mixed with the 4 plasmids without an exchange medium on the second day. Adherent 293 T cells were packaged lentiviral as previously described [21]. 5 × 10^6^ adherent 293 T cells were seeded in a 100 mm dish a day in advance, mixed with the 4 plasmids on the second day, and added 5 mL complete medium on the third day.

After 72 hours, the cells suspension were harvested for ZOE™ Fluorescent Cell Gallery to verify green fluorescent protein expression. Intact cells removed by low-speed centrifugation (300 g for 5 min) and then centrifuged at high-speed centrifugation (3200 g for 30 min) to discard larger particles. 100 µL of the supernatants was used in the HIV p24 Antigen Rapid Test Cassette (Botelong Immunotechnologies Co., Ltd., Suzhou, China) to determine the packaging effect. To begin transduction, we inoculated 6-well plates with adherent 293 T cells at a total number of 5.0 × 10^5^ cells followed that with 0–64 µL of viral supernatant and 2 mL of polybrene (4 g/mL). Three days later, GFP was used to detect the viral packaging ability of adapted 293 T suspension cells by flow cytometry (BD Biosciences). Wells with fewer than 1–10% GFP positive cells were chosen to calculate the transduction titer (TU/mL) using the following formula: (total transduced cell number × proportion of positive cells)/(viral supernatant volume) [22,23].

### Cryopreservation solution selection

293 T suspension cells can grow and pack LV in serum-free culture. Cryopreservation should be used with a serum-free formula to ensure consistency across the process. Cryopreservation solutionI was 90% v/v FBS plus 10% DMSO, cryopreservation solutionII was 90% v/v human serum albumin (HAS, CSL Behring AG, Switzerland) plus 10% DMSO, cryopreservation solutionIII was 65% v/v F medium plus 10% DMSO and 25% HAS, and cryopreservation solutionIV was 90% v/v F medium plus 10% DMSO. Cells were counted, centrifuged, and discarded the supernatant. Then, the cell was suspended with four cryopreservation solutions, and each in three tubes. The final concentration per cryotube was 1 ×10^7^ cells/mL. The tubes were held at −80°C overnight in a Mr. Frosty apparatus (Thermo Scientific) and then transferred to liquid nitrogen for a month or more [24]. After resuscitation, the effects of these different cryopreservation solutions on cell morphology, viability, and growth were observed by microscope. The best cryopreservation solution was selected and frozen for 293 T suspension cells and a seed bank was established for late verification tests or large-scale production. LV packaging experiment was repeated at least three times using three different batches of the frozen cell.

### Bioreactor cultivation

We used a 15-L 3-impeller agitating bioreactor (Z4AEZ0015M, Applikon Biotechnology, Netherlands) with a working volume of 5.5 L containing 0.1% (v/v) nonionic surfactant Pluronic F-68 (Sigma-Aldrich), a constant 130 rpm stirring speed, and 50% dissolved oxygen level. Air was applied to the surface while air and CO_2_ bubbled up from the bottom. When the dissolved oxygen fell below 50%, oxygen was pumped in from the bottom. To test for aseptic conditions and reactor airtightness, 24 hours before beginning the experiment, we pumped a portion of a fresh medium into the reactor. Once the experiment began, a constant temperature of 37°C, and a pH value of 7.1 were maintained [25]. Both fermentation and transfection conditions were followed the flask shaking system to pack LV in the bioreactor.

Plasmid complex was added at 10% of the working volume when cell density reached a range of 1.0 × 10^6^ cells/mL. After 24 hours post-transfection, 5% of the working volume of supplemented medium was added. The culture supernatants were harvested at 48 hours post-transfection by ultracentrifugation according to the above operation in LV packaging verification and then allowed to infect primary T cells with a multiplicity of transduction (MOI) of 0, 0.2, 2, 20, and 200. The volume of viral supernatant required was calculated according to the formula [26]: MOI = (transduction titer × viral supernatant volume)/cell number. According to previously described methods, the T-cells have acquired from volunteers who participated in this study, agreed and signed Informed Consent and transfected [27]. Three days after transduction, the cells were used for flow cytometry to evaluate the transduction efficiency.

### Statistics

The data was representative of three independent experiments, and GraphPad Prism 6 (GraphPad Software Inc., La Jolla, CA, USA) was used for statistical analysis. The data was presented as the mean ± standard deviation. The comparison between groups was done by a two-way analysis of variance test followed by Tukey’s test. Values less than 0.05 were considered to be statistically significant and p values <0.05 or <0.01 were indicated by * or **, respectively.

## Results

To establish large-scale processes for functional LV production in a stirred bioreactor without micro-carriers, we adapted HEK293T adherent cells to suspension cells in a serum-free medium, plotted with the growth curve, and validated the virus packaging abilities. Eventually, the cells were stored in an effective solution and applied to a bioreactor for LV production.

### Adherent cell cultivation in serum-free medium

**Figure 1.**
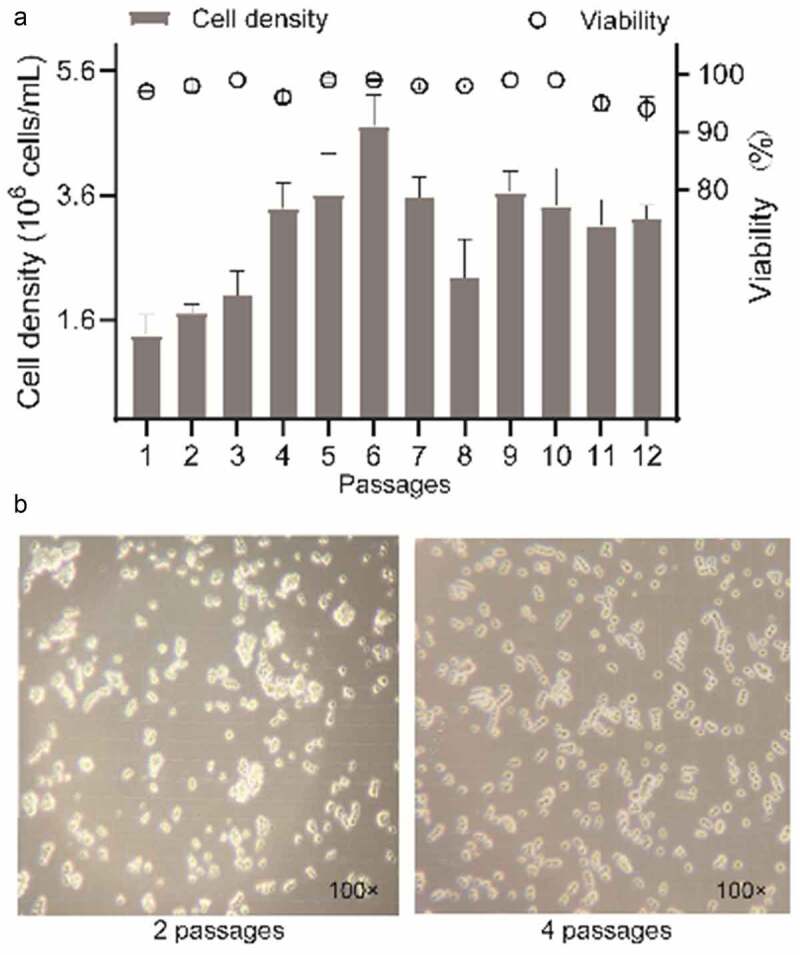
**293 T suspension cells grown in shake flasks in F medium during cell adaptation**. (a) the percentage of cell growth and viability. (b) cell morphology without shaking was observed using a 100× bright field microscope and images were captured using a cell phone camera

Figure 1a shows that after 12 subcultures, cell viability of more than 90% was maintained, while adaption cell density expanded from 4.0 × 10^5^ cells/mL to 1.7 ± 0.4 × 10^6^–4.7 ± 0.5 × 10^6^ cells/mL after 3 days in culture except for the first passages. The second adapted generation of 293 T suspension cells had a relatively high rate of cell aggregation (Figure 1b). After two passages, the cells were single-celled or formed chains with uniform size and brightness. Suspension 293 T cells in F medium mostly remained single, with aggregates of up to 5 cells in Figure 1b.

### Effect of inoculum size on the growth curve of 293 T suspension cell

**Figure 2.**
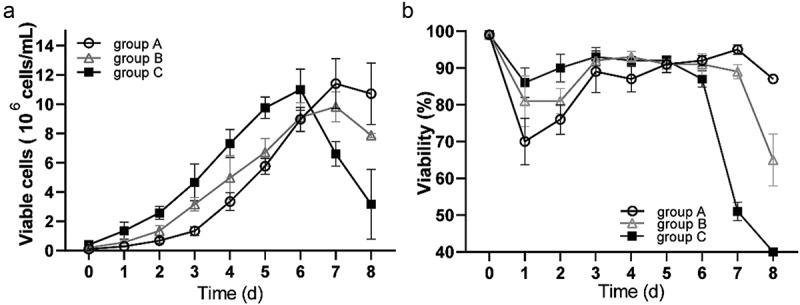
**Growth results over 8 d of adapted suspension 293 T cells with cell densities of 1.0 × 10^5^ cells/mL (group A), 2.0 × 10^5^ cells/mL (group B), and 4.0 × 10^5^ cells/mL (group C), respectively**. (a) live cell growth curve; (b) cell viability percentages. data are means (SD) of 3 experiments

The growth parameters of three different incubated cell concentrations were examined to determine the effect of inoculum size on the growth curve of 293 T suspension cells: 1.0 × 10^5^ cells/mL (group A), 2.0 × 10^5^cells/mL (group B), and 4.0 × 10^5^ cells/mL (group C). Initially, all groups experienced increasing viable cell density, but group C began decreasing on day 6, and groups A and B began decreasing on day 7 (Figure 2a). The percentage of cell viability was consistent, and group C had relatively little influence on cell viability in the early stages of culture (Figure 2b). These experimental results demonstrated that the 20 mL F medium met basic nutritional requirements of 293 T suspension cells cultured at high inoculation density (group C) for 6 days.

### Suspension cell virus packaging abilities

**Figure 3.**
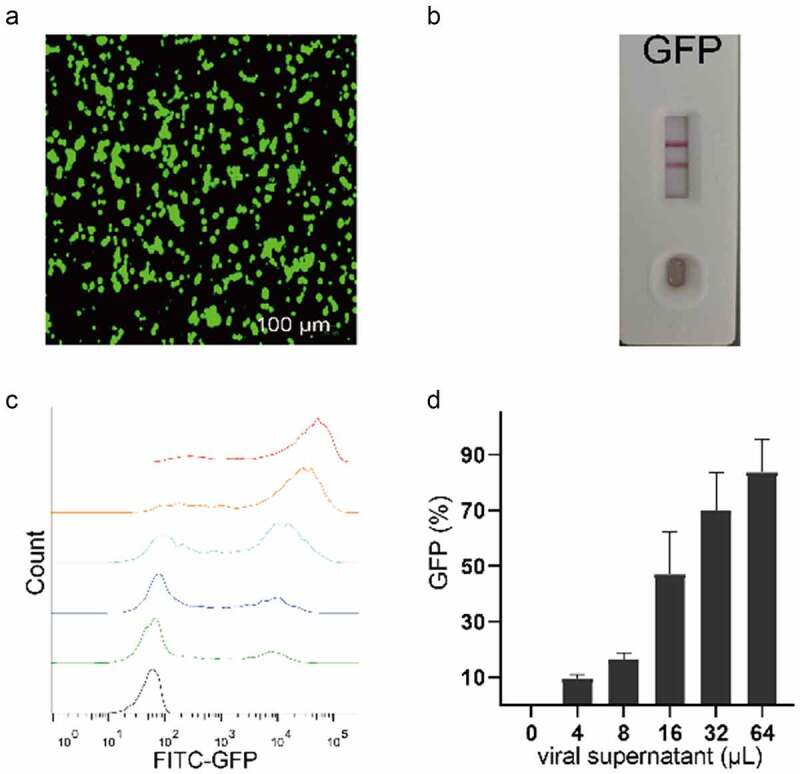
**Verification of virus packaging capability of 293 T suspension cells**. (a) observation of GFP expression in 293 T suspension cells after infection using 100 µm for green field microscopy. (b) 100 µL cell supernatant was collected, treated at 72 h post-transduction, and tested using an HIV p24 Antigen Rapid Test Cassette. the test shows positive results (2 horizontal lines). (c, d) inoculated 293 T cells with 6 increasing amounts (0–64 µL) of the supernatant were detected using a flow cytometer

The expression plasmid with GFP was used to verify whether 293 T suspension cells could pack the LV. Fluorescent results in Figure 3a suggested that the cells were able to activate GFP expression. After centrifugation and filtration, the protein card test verified positive results (Figure 3b). The rate of GFP positive cells in infected cells increased in step with the increasing volume of the transfected virus and the virus titer (1.2 × 10^7^ TU/mL) was calculated as the GFP positive cells with about 9.6 ± 1.3% conversion (Figure 3c and 3d). These results indicated that when 293 T adherent cells were adapted into suspension cells, and they maintained virus replication ability.

### Cryopreservation solution selection

**Figure 4.**
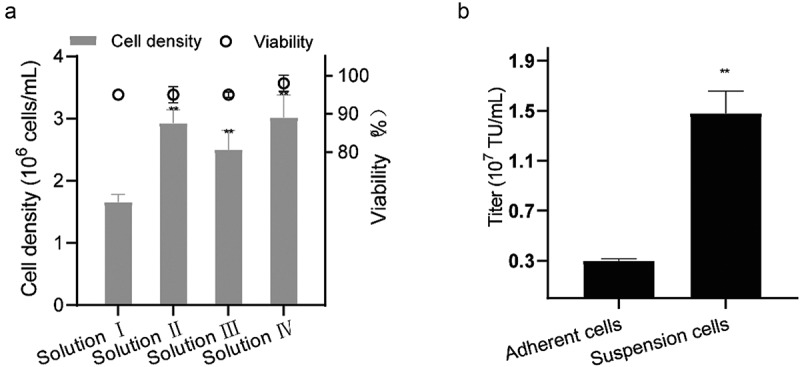
**Effect of thawing on 293 T suspension cells**. (a) Viabilities and densities of 293 T suspension cells grown after freezing in 4 solutions at the first passage and then thawing. **P < 0.01. (b) the titer of the resuscitated adherent and suspension 293 T cells packing the LV. data are means (SD) of 3 experiments

When we tested the four cryopreservation solutions for suspended 293 T culture, we found that the cell of solution IV grew from 4.0 ± 0.1 × 10^5^ cells/mL to 3.0 ± 0.4 × 10^6^ cells/mL cell densities with cell viability of 98.0 ± 2.0%, being the highest values for all 4 solutions after 3 days of cultivation (Figure 4a). Therefore, adding 10% DMSO directly to the culture medium for cryopreservation not only reduced protein involvement and process cost but also aided cryopreservation. Figure 4b and Supplemental material Table S2 show that after cells were resuscitated, the suspension cells still had an average 1.5 ± 0.2 × 10^7^ TU/mL virus packaging capability in shake-flasks while adherent cells just had 0.3 ± 0.0 × 10^7^ TU/mL virus. This represented a nearly five-fold improvement in virus packaging capability over the original adherent 293 T cells.

### Bioreactor cultivation and LV packaging

**Figure 5.**
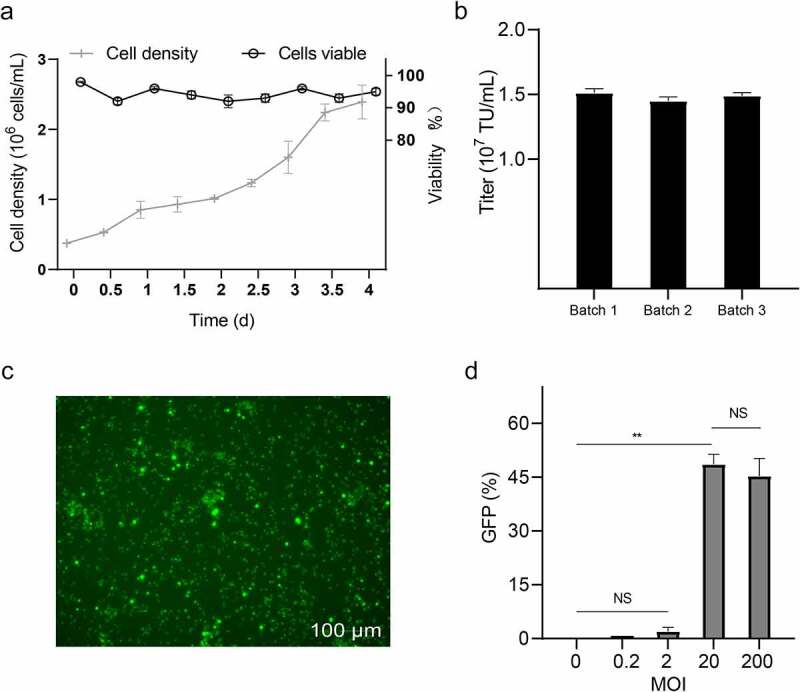
**Verification of the virus packaging capability of 293 T suspension cells in a 5.5 L working volume bioreactor**. (a) the percentage of cell growth curve and viability during fermentation; (b) titer of the 293 T suspension cells packing the LV data are means (SD) of 3 experiments. (c) observation of GFP expression in T cells after transduction using 100 µm for green field microscopy. (d) inoculated T cells at a multiplicity of transduction (MOI) of 0, 0.2, 2, 20, and 200 viruses were detected using a flow cytometer. data are means (SD) of 3 experiments. **P < 0.01, ^NS^*P* ˃ 0.05

The adapted 293 T cells also need to be proved to suitable for cultivation in the bioreactor. After 2 days of fermentation at a seeding density of 3.8 ± 0.4 × 10^5^ cells/mL (130 rpm, 50% OD, pH 7.1, and 37°C), cell viability was greater than 90% and the growth rate had increased to 1.0 ± 0.1 × 10^6^ cells/mL, indicating that suspension cells could grow in a bioreactor (Figure 5a). The titer of LV, detected by flow cytometry (Figure 5b) at 72 hours post-transduction of adherent 293 T cells, was about 1.5 ± 0.3 × 10^7^ TU/mL. These results showed that cultured suspension cells could grow in the bioreactor and pack LV at a titer similar to that of flask transduction. Low MOI of the unpurified and unconcentrated LV had suffered from very low transduction efficiency to T cells, but transduction efficiency was 48 ± 0.3% (*P* < 0.01) after MOI = 20 (Figure 5c and 5d).

## Discussion

Cell adaptation to suspension growth was not always as straightforward and successful as previously described [28]. A stepwise reduction of FBS percentage was performed to adapt serum-free culture conditions, and it usually took at least 4–8 weeks [10,14]. More recent studies have confirmed that specific cell lines may also change their expression of surface receptors during long-term adaptation, which then affects production purposes [29]. Compared to commercially available 293 T suspension cells, the 293 T cell line used in this experiment are from a stable cell source with non-gene editing, convenient for producing biomedical products and clinical applications. 293 T adherent cells successfully were adapted the into suspension cells for about a week in this study using the commercial serum-free medium 293 CD05 and SMM293-TII .

Our method also has advantages over other adaptation methods, such as easy cultivation, no additional anti-agglomerating agents [30], no need for genetic modification, and micro-carriers to stay uniformly dispersed proliferation. The proposed method saves time, by markedly reducing the introduction of exogenous impurities, being more appropriate for the clinical production of biological products than previously reported adaptation processes. Our adapted cells have passed a sterility test and relevant institutions have identified bacteria. Both seed and a working bank have been established for large-scale production and future utilization.

Lentiviral packaging required 3–5 days [22,23]. The suspension cells with an inoculation density of 4 × 10^5^ cells/mL resulted in better viability and proliferation and were nutritionally depleted until 6 days. Hence, it could be used as the inoculum cell density. After 3 d of cultivation, cell density had reached at least 3.8 times. Cells could keep their susceptibility and permissiveness to allow efficient virus replication [31]. After multiple passages and cryopreservation recovery, the suspension cells had proliferated and maintained their ability to package functional virus. Therefore, we have completed the critical process steps to enable a large-scale and clinical LV production process in a relatively short period.

Suspension cultures are amenable for process automation and easy regulation and control of optimized conditions, resulting in being the current choice for most large-scale bio-manufacturing applications in bioreactor [32]. Today, a variety of stirred-tank bioreactors with well-characterized hydrodynamic properties are available for cell culture [33]. However, the bioreactor demonstrated a deficiency in shear forces and oxygen transfer rates [28]. This study shows that the 15-L and 3-impeller agitating bioreactor can be used to culture human 293 T cells at high-density and package functional LV, which infects T cells without purification and concentration treatments, with excellent results. This was crucial to the feasibility of the product for later optimization.

Suspension cells in a 5.5-L working volume of the bioreactor can be produced about 1.5 ± 0.3 × 10^7^ TU/mL LV stock solution. The production period was just about 4 days. The adapted 293 T suspension cells were successfully cultivated in the bioreactor with a reasonable yield of LV, which means this process can be easily scaled up for industrial production. This yield may not be directly compared between studies because of the difference in transient transfection method, virus determination method, the size of expressed genes, and downstream processing [34,35]. However, as a preliminary study, it has a nearly five-fold improvement in virus packaging capability over the original adherent cells and we believe further improvements of the yield can be made by continuing to optimize the technological process, such as cultivation, transfection, and purification process.

## Conclusions

In this study, we established an efficient method with one week of adaption and without the cost of micro-carriers but also has wholly done serum-free process from adaption to cryopreservation, shake flask to scale-up bioreactor as well as the utility of this functional virus, which laid the foundation for further scale-up and eventual industrial application. This study also has significance as a reference for the development of similar 293 T-derived produce in the future and will primarily reduce costs for CAR-T’s clinical and commercial development.

## Supplementary Material

Supplemental Material

## Data Availability

The data used to support the findings of this study are available from the corresponding author upon request.
