# Supplementary material for: Establishing functional lentiviral vector production in a stirred bioreactor for CAR-T cell therapy
Source: Bioengineered. 2021 May 28;12(1):2095–105. doi: 10.1080/21655979.2021.1931644 (PMC8806440; doi:10.1080/21655979.2021.1931644)
Supplement: Supplemental Material [file KBIE_A_1931644_SM7099.docx]

Supplemental material

Table S1 Comparison of different medium on growth speed and virus titers.

| No. | Medium | Rapid adaptation | Growth speed | Rank in Virus yields |
| --- | --- | --- | --- | --- |
| A | OPM-293 CD05 Medium | Yes | Fast | 2 |
| B | HEK293 Serum-free Medium | No |  |  |
| C | SMM293-TⅡ | Yes | Low | 5 |
| D | HEK293 CD Medium | No |  |  |
| E | KOP293 Serum-free Medium | Yes | Middle | 7 |
| F | A+D | Yes | Middle | 1 |
| G | A+E | Yes | Middle | 3 |
| H | D+E | Yes | Middle | 6 |
| I | A+D+E | Yes | Middle | 4 |

Table S2 Comparison of different culture on adherent and suspension cells.

| 293 cell | Count (cells) | Culture before transfection(h) | | 24 h Supplement(mL) | Volume（mL） | Titer  (TU/mL) |
| --- | --- | --- | --- | --- | --- | --- |
| Adherent | 5×10^6^ | 20 | 5 | | 15 | 0.3×10^7^ |
| Suspension | 2×10^7^ | 0 | 1 | | 20 | 1.5×10^7^ |
